# Metabolic Effects of Glucose-Fructose Co-Ingestion Compared to Glucose Alone during Exercise in Type 1 Diabetes

**DOI:** 10.3390/nu9020164

**Published:** 2017-02-21

**Authors:** Lia Bally, Patrick Kempf, Thomas Zueger, Christian Speck, Nicola Pasi, Carlos Ciller, Katrin Feller, Hannah Loher, Robin Rosset, Matthias Wilhelm, Chris Boesch, Tania Buehler, Ayse S. Dokumaci, Luc Tappy, Christoph Stettler

**Affiliations:** 1 Department of Diabetes, Endocrinology, Clinical Nutrition and Metabolism, Inselspital, Bern University Hospital, University of Bern, 3010 Bern, Switzerland; lia.bally@insel.ch (L.B.); patrick.kempf@insel.ch (P.K.); t.zueger@bluewin.ch (T.Z.); christian.speck@outlook.com (C.S.); nicola.pasi@students.unibe.ch (N.P.); katrin.feller2@insel.ch (K.F.); hannah.loher@bluewin.ch (H.L.); 2 Department of Radiology, University Hospital Centre and University of Lausanne, 1011 Lausanne, Switzerland; ciller@gmail.com; 3 Centre for Biomedical Imaging (CIBM), Signal Processing Core, 1015 Lausanne, Switzerland; 4 Department of Physiology, Faculty of Biology and Medicine, University of Lausanne, 1005 Lausanne, Switzerland; robin.Rosset@unil.ch (R.R.); luc.tappy@unil.ch (L.T.); 5 Department of Cardiology, Interdisciplinary Center for Sports Medicine, Inselspital, Bern University Hospital, University of Bern, 3010 Bern, Switzerland; matthias.wilhelm@insel.ch; 6 Department of Clinical Research and Department of Radiology, University of Bern, 3010 Bern, Switzerland; chris.boesch@insel.ch (C.B.); buehler_tania@gmx.net (T.B.); ayse.dokumaci@insel.ch (A.S.D.)

**Keywords:** carbohydrates, glucose, fructose, type 1 diabetes, exercise, glycaemia, substrate oxidation

## Abstract

This paper aims to compare the metabolic effects of glucose-fructose co-ingestion (GLUFRU) with glucose alone (GLU) in exercising individuals with type 1 diabetes mellitus. Fifteen male individuals with type 1 diabetes (HbA1c 7.0% ± 0.6% (53 ± 7 mmol/mol)) underwent a 90 min iso-energetic continuous cycling session at 50% VO_2max_ while ingesting combined glucose-fructose (GLUFRU) or glucose alone (GLU) to maintain stable glycaemia without insulin adjustment. GLUFRU and GLU were labelled with ^13^C-fructose and ^13^C-glucose, respectively. Metabolic assessments included measurements of hormones and metabolites, substrate oxidation, and stable isotopes. Exogenous carbohydrate requirements to maintain stable glycaemia were comparable between GLUFRU and GLU (*p* = 0.46). Fat oxidation was significantly higher (5.2 ± 0.2 vs. 2.6 ± 1.2 mg·kg^−1^·min^−1^, *p* < 0.001) and carbohydrate oxidation lower (18.1 ± 0.8 vs. 24.5 ± 0.8 mg·kg^−1^·min^−1^ *p* < 0.001) in GLUFRU compared to GLU, with decreased muscle glycogen oxidation in GLUFRU (10.2 ± 0.9 vs. 17.5 ± 1.0 mg·kg^−1^·min^−1^, *p* < 0.001). Lactate levels were higher (2.2 ± 0.2 vs. 1.8 ± 0.1 mmol/L, *p* = 0.012) in GLUFRU, with comparable counter-regulatory hormones between GLUFRU and GLU (*p* > 0.05 for all). Glucose and insulin levels, and total glucose appearance and disappearance were comparable between interventions. Glucose-fructose co-ingestion may have a beneficial impact on fuel metabolism in exercising individuals with type 1 diabetes without insulin adjustment, by increasing fat oxidation whilst sparing glycogen.

## 1. Introduction

The beneficial effects of exercise on cardiovascular health and general well-being in patients with type 1 diabetes are well documented [1,2], however, maintaining glycaemic control during exercise remains complex and demanding. The use of exogenous insulin which leads to supraphysiological peripheral insulin levels, in addition to the exercise-induced increase in muscle glucose uptake, predisposes to hypoglycaemia (e.g., dangerous fall in blood glucose levels, below the normal physiological range). Consequently, fear of exercise-related hypoglycaemia is an important factor deterring patients with type 1 diabetes from exercising, despite the fact that many of these individuals are physically fit and wish to be active [3]. 

Current exercise management guidelines provide pragmatic recommendations, such as adjusting insulin doses and/or increasing the amount of carbohydrates (CHO) ingested before, during, or after exercise [4]. However, simple reduction of insulin dosages require pre-planning and may not always achieve desired results [5]. Several studies have evaluated the dosing and administration schedules of CHO to mitigate against hypoglycaemia [6,7]. The metabolic effects of differing types of CHO in the context of physical activity, however, remain under-studied, particularly in individuals with type 1 diabetes. Although the glycaemic effects of different types of CHO under conditions of insulin dose adjustment have been studied [8,9,10,11], the metabolic responses under usual insulin dose conditions remain unclear.

Fructose is a monosaccharide with a considerably different metabolism from that of glucose. Orally-ingested fructose is absorbed via specific intestinal transporter (GLUT5) [12] and is then almost completely extracted by the liver and metabolized by a specific set of enzymes [13]. Fructose, as a subsidiary energy source, is converted to primary energy substrates such as lactate, glucose, and lipids which can either be released into circulation and used by other organs (e.g., the exercising muscle) or stored in the liver [13]. Fructose may therefore act as an alternative to glucose in meeting energy requirements, without the need for insulin, thereby being of particular interest to patients with type 1 diabetes. While the co-ingestion of glucose and fructose under exercise conditions has been previously investigated in healthy non-diabetic individuals [14,15,16], there is no systematic analysis to date in patients with type 1 diabetes. Therefore, we aimed to investigate the effects of glucose-fructose co-ingestion (GLUFRU) compared to glucose alone (GLU) in exercising type 1 diabetes individuals, on glycaemic stability and exercise-associated metabolism. 

## 2. Material and Methods

### 2.1. Inclusion Criteria

Fifteen recreationally active male adults with well-controlled type 1 diabetes were recruited for this study. Eleven participants were insulin pump users and four were multiple daily injection users. Volunteers were eligible if they had undetectable C-peptide (<100 pmol/L with concomitant blood glucose ≥4 mmol/L), had no known diabetes-related complications, were on stable insulin regime for at least three months prior to the study, and were not on any medications other than insulin. All participants signed informed consent prior to the start of study-related procedures. The study was approved by the Ethics Committee Bern (KEK 001/14). 

### 2.2. Experimental Design and Protocol

This was a prospective non-randomised cross-over design study. Baseline study visits included indirect calorimetry to determine basal metabolic rate (BMR), bioimpedance analysis for lean body mass calculation (BIA 101, Akern, Pontassieve FI, Italy), and a stepwise incremental exercise test on a bicycle ergometer with breath-to-breath spiroergometry (Cardiovit AT-104 PC; Schiller, Baar, Switzerland), as previously described [17]. 

A 1:1 glucose-fructose mixture (GLUFRU) or glucose alone (GLU) was given orally to maintain stable glycaemia over a 90 min cycling session at 50% VO_2max_. GLUFRU and GLU consisted of 20% (1:1 glucose-fructose) and 10% CHO solution (Glucosum monohydricum, Hänseler AG, Herisau, Switzerland, D-Fructose, Fluka Analytic, Sigma Aldrich, Buchs, Switzerland), respectively. The concentration of GLUFRU solution was higher compared to GLU to ensure that both solutions provided comparable immediate glycaemic effects at the same volume. GLUFRU or GLU were provided based on personalised CHO-intake regimens which were pre-determined during the familiarisation period (90 min cycling session) in order to account for variable individual responses. The primary outcome of the study was the CHO requirements to maintain stable glycaemia during the final 30 min of the exercise period. Secondary endpoints included assessment of whole body substrate oxidation, glucose turnover, as well as measurements of metabolites and hormones. Ten participants underwent GLU first, followed by GLUFRU. Conversely, five participants underwent GLUFRU first, followed by GLU. 

### 2.3. Pre-Study Standardization Procedures 

Participants consumed a standardized diet with a pre-defined daily CHO quantity corresponding to 50% of their calculated energy expenditure 48 h before the main study intervention day. The diet was replicated prior to the second intervention. Foods naturally-enriched in ^13^C-CHO were avoided to limit baseline shifts in expired ^13^CO_2_ [18]. Participants were additionally requested to avoid strenuous exercise (pedometer record <5000 steps per day), alcohol, and caffeine. Patients adhered to their usual insulin regime, and daily glycaemia levels were assessed using a continuous glucose monitor (CGM) and capillary glucose measurements. On the main study day, participants were given a standardized breakfast containing one-sixth of each individual’s estimated daily CHO amount at 0700, for which participants bolused according to their carbohydrate-to-insulin ratio. Participants were admitted to the research facility at 0930. Basal insulin delivery was not adjusted prior to exercise, and kept identical during both study interventions. 

### 2.4. Sampling Procedures for Metabolites and Hormones

An 18 G cannula was inserted into the antecubital vein of each forearm upon arrival to the research facility. Blood glucose was measured every 10 min (YSI 2300; Yellow Springs Instruments, Yellow Springs, OH, USA). Blood sampling for insulin, counter-regulatory hormones (catecholamines, growth hormone (GH), glucagon, cortisol) and metabolites (lactate, non-esterified fatty acids (NEFAs)) was performed 50 min prior to exercise, at 10, 30, 60, and 80 min of exercise, as well as in the recovery phase (120 min after exercise completion). Insulin, GH, and cortisol were measured using commercially available immunoassay kits (insulin: Architect, Abbott, Baar, Switzerland; GH: Immulite, Siemens, Zurich, Switzerland; cortisol: Modular, Roche, Rotkreuz, Switzerland). Glucagon was measured using a double radioimmunoassay (Siemens, Zurich, Switzerland) in ethylenediaminetetraacetic acid (EDTA) plasma mixed with aprotinin, immediately cooled and frozen after separation. NEFA levels were assessed using a kit from Wako Chemicals (Dietikon, Switzerland). Lactate and pH were determined electrochemically using the ABL 835/837 FLEX (Radiometer, Thalwil, Switzerland) analyser. Plasma catecholamines were quantified using ultraperformance liquid chromatography-tandem mass spectrometry (Waters Acquity UPLC/TQD, Manchester, UK) [19]. 

### 2.5. Respiratory Gas Exchange, Cardiopulmonary Monitoring, and Substrate Oxidation

VCO_2_ and VO_2_ were measured immediately before, during, and 120 min after exercise completion. The 90 min exercise session involved six spirometric recording phases, each performed over 5 min periods at 15, 35, 55, 65, 75, and 85 min of exercise. Net substrate oxidation and energy expenditure were calculated from standard indirect calorimetry equations [20].

Heart rate was recorded continuously by a portable three channel electrocardiogram (ECG) (Lifecard CF, Del Mar Reynolds Medical Inc., Irvine, CA, USA). Rate of perceived exertion (RPE) was assessed by the Borg scale every 10 min. 

### 2.6. Stable Isotopes

Orally supplied CHO solutions were labelled with 0.5% U-^13^C_6_-fructose for GLUFRU and 0.5% U-^13^C_6_-glucose for GLU. 6,6-^2^H_2_-glucose (Cambridge Isotope Laboratories, Tewksbury, MA, USA) was infused for both interventions. Double background enrichment measurements (blood and breath samples) were taken immediately after intravenous cannulation. Twenty minutes before exercise, a primed (0.6 mg·kg^−1^(mmol/L)^−1^) constant infusion of 30 μg 6,6-^2^H_2_-glucose kg^−1^·min^−1^ was initiated. At the onset of exercise, the 6,6-^2^H_2_-glucose infusion rate was quadrupled to minimize changes in enrichment [21]. Blood and breath samples were obtained during exercise at 59, 69, 79, and 89 min. Plasma 6,6-^2^H_2_-glucose and ^13^C-glucose isotopic enrichment (IE) were measured using gas-chromatography mass-spectrometry (GC-MS) (Hewlett-Packard Instruments, Palo Alto, CA, USA) in chemical ionization mode, as previously described [22]. ^13^CO_2_-IE was measured by isotope-ratio mass spectrometry (SerCon, Crewe, UK). Plasma fructose concentrations were measured using a previously published protocol from Petersen and colleagues [23]. 

### 2.7. Calculations of Glucose and Fructose Turnover

Glucose and fructose turnover were computed during the last 30 min of exercise, to ensure plateau enrichment was achieved. The rate of glucose appearance (R_a_) and disappearance (R_d_) were calculated from 6,6-deuterated glucose dilution using Steele’s equation for non-steady state conditions, assuming an effective fraction of 0.65 and a distribution volume of 0.22 L/kg [24]. Glucose metabolic clearance rate (MCR) was calculated as R_d_/glucose concentration. Muscle glycogen oxidation was estimated as the difference between net CHO oxidation and glucose R_d_, assuming that 100% of plasma glucose uptake was oxidized during exercise [25]. Gluconeogenesis from fructose was assessed by the product of R_a_ and isotope enrichment ratio of plasma glucose (M + 3) and ingested fructose (M + 6). The ratio of ^13^C-abundance in the expired air and ingested glucose and fructose, using a recovery factor of 1.0, provided an estimate of the oxidised amount of fructose (GLUFRU) and glucose (GLU) [26]. A bicarbonate correction factor, assuming a bicarbonate pool of 14.2 mmol/kg, was applied to estimate ^13^C-fructose/glucose oxidation [27]. 

### 2.8. Statistical Analysis

We estimated that twelve participants would provide 95% power to detect a mean difference of 8.2 g of glucose given during the last 30 min, at a level of 5% significance [15]. Assuming a SD of 7 g (based on previous metabolic studies in participants with type 1 diabetes with inherent glycaemic variability [28]), we estimated a sample size of 12, which was increased to 15 to account for drop outs (related to the complexity of study procedures and visits). Data were analysed using Stata 13.0 (Stata Corporation, College Station, TX, USA), Matlab R2015a (The MathWorks, Inc., Natick, MA, USA), and GraphPad Prism software 5.0 (GraphPad Software Inc., San Diego, CA, USA). Differences in hormones, metabolites, and substrate oxidation were evaluated using paired comparisons of area under the curve. Glucose turnover was compared using values obtained during the last 30 min of exercise. Continuous variables were analysed for normality using the Shapiro-Wilk test and qq-plots. Student’s paired t tests were used to identify differences for normally distributed variables, and Wilcoxon’s signed rank tests were used for non-normally distributed variables. A *p*-value <0.05 was considered statistically significant. Values are expressed as mean ± standard error of the mean (SEM), unless otherwise specified. 

## 3. Results

### 3.1. Baseline Characteristics and Pre-Study Conditions

Baseline characteristics are shown in Table 1. Total daily CHO intake and insulin dosage were similar for GLUFRU and GLU during the 48 h prior to the main study intervention (CHO: 353 ± 13 vs. 351 ± 11 g/day, *p* = 0.83; insulin dose: 71 ± 4 vs. 70 ± 3 U/day, *p* = 0.49). 

### 3.2. CHO Requirements 

**Figure 1:**
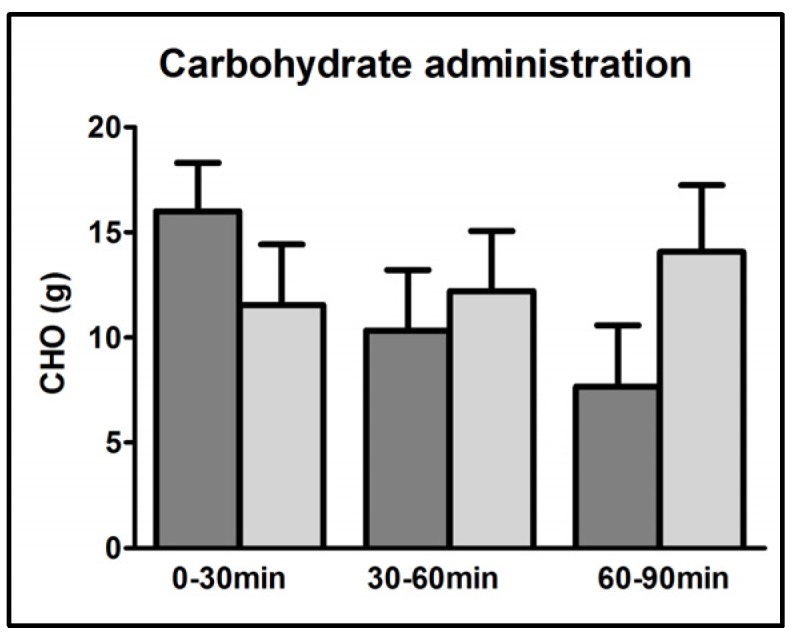
Carbohydrate administration during first, second, and third 30 min-intervals of exercise. GLUFRU (glucose-fructose co-ingestion) = dark grey bar and GLU (glucose alone ingestion) = light grey bar. Results are expressed as mean ± SEM.

The primary endpoint (CHO requirements within the last 30 min of exercise) did not differ significantly between interventions: 7.7 ± 2.9 vs. 14.1 ± 3.2 g (*p* = 0.14) in GLUFRU and GLU, respectively. Total CHO requirements to maintain stable glycaemia over the whole exercise period were similar (34.0 ± 2.9 g in GLUFRU and 37.8 ± 5.3 g in GLU, *p* = 0.46). Notably, half of the supplied CHO in GLUFRU consisted of fructose (17 g) (Figure 1). 

### 3.3. Energy Expenditure 

Total energy expenditure during exercise was 3.3 ± 0.2 and 3.2 ± 0.1 MJ (*p* = 0.34), in GLUFRU and GLU, respectively. Average heart rate did not differ between GLUFRU and GLU (138.6 ± 3.1 vs. 133.4 ± 3.1 beats per minutewhich corresponded to 73% ± 2% and 71% ± 2% of maximal heart rate, respectively (*p* = 0.12). The measured oxygen consumption was similar during both GLUFRU (24 ± 1 mg·kg^−1^·min^−1^) and GLU (23 ± 1 mg·kg^−1^·min^−1^), which corresponded to 53% ± 2% VO_2max_ and 51% ± 3% VO_2max_, respectively (*p* = 0.20).

### 3.4. Glycaemia and Insulin Levels

**Figure 2:**
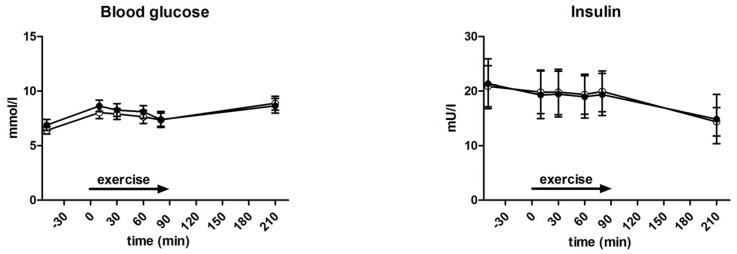
Measured blood glucose and insulin during GLUFRU (black circle) and GLU (white circle). Left to right: blood glucose, *p* = 0.67; insulin, *p* = 0.89. Results are expressed as mean ± SEM.

During exercise, glucose (8.1 ± 0.3 vs. 7.7 ± 0.2 mmol/L, *p* = 0.67) and insulin (138.3 ± 0.8 vs. 141.8 ± 0.9 pmol/L, *p* = 0.23) levels were not significantly different between GLUFRU and GLU (Figure 2). Insulin levels 120 min after exercise were 106.7 ± 32.6 pmol/L in GLUFRU and 103.2 ± 18.7 pmol/L in GLU (*p* = 0.89). Corresponding glucose levels were 8.7 ± 0.7 and 8.9 ± 0.6 mmol/L for GLUFRU and GLU, respectively (*p* = 0.72) (Figure 2).

### 3.5. Metabolites and Counter-Regulatory Hormones 

**Figure 3:**
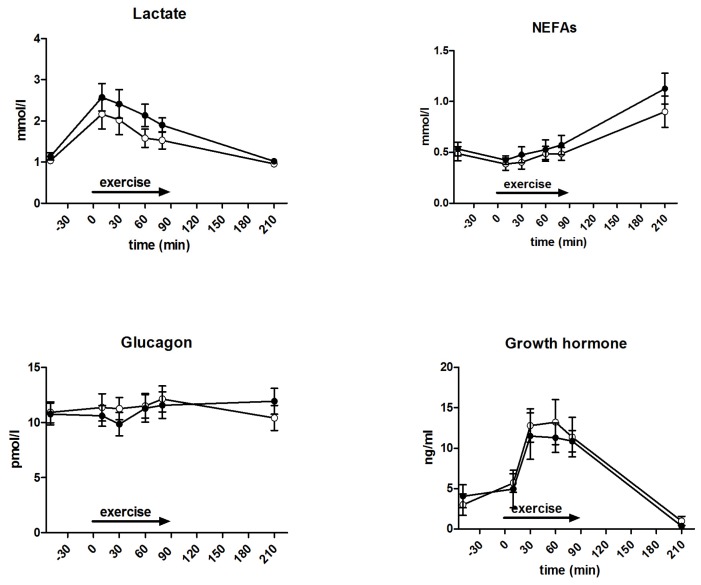

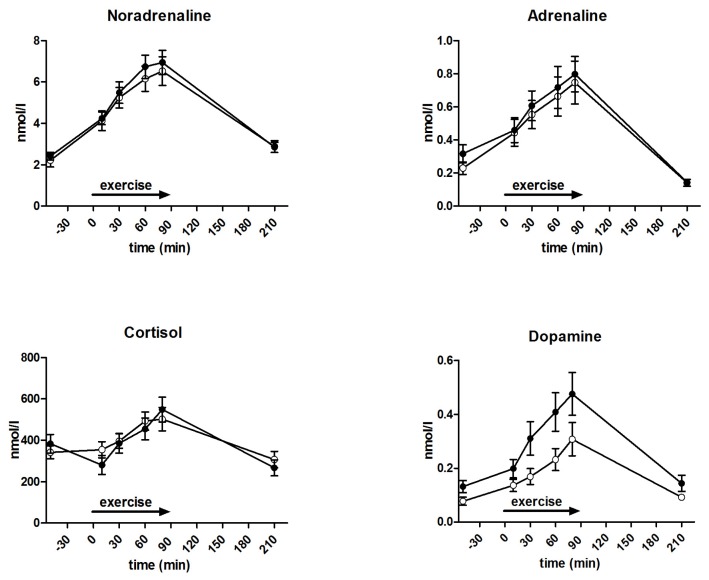
Measured hormones and metabolites during GLUFRU (black circle) and GLU (white circle). Clockwise from top left: lactate, *p* = 0.012; non-esterified fatty acids (NEFAs), *p* = 0.43; growth hormone, *p* = 0.50; adrenaline, *p* = 0.39; dopamine, *p* = 0.037; cortisol, *p* = 0.54; noradrenaline, *p* = 0.45; glucagon, *p* = 0.16. Results are expressed as mean ± SEM.

Measured metabolites and hormones are shown in Figure 3. During exercise, lactate levels were significantly higher (2.2 ± 0.2 vs. 1.8 ± 0.1 mmol/L, *p* = 0.012), but not at 120 min after exercise completion (*p* = 0.58), in GLUFRU compared to GLU. No differences in pH levels were observed between GLUFRU and GLU (7.39 ± 0.01 vs. 7.38 ± 0.00, *p* = 0.33). NEFA levels during exercise were 0.5 ± 0.04 mmol/L in GLUFRU and 0.4 ± 0.03 mmol/L in GLU (*p* = 0.43). During exercise, mean glucagon levels were 10.7 ± 0.6 and 11.6 ± 0.6 pmol/L (*p* = 0.16) in GLUFRU and GLU, respectively. Mean GH levels (9.7 ± 1.1 vs. 10.8 ± 1.1 ng/mL, *p* = 0.50), noradrenaline (5.9 ± 0.3 vs. 5.5 ± 0.3 nmol/L, *p* = 0.45), adrenaline (0.6 ± 0.1 nmol/L vs. 0.6 ± 0.1 nmol/L, *p* = 0.39), and cortisol (417.6 ± 28.2 and 436.8 ± 23.1 nmol, *p* = 0.54) were comparable between GLUFRU and GLU. Dopamine levels were significantly higher in GLUFRU compared to GLU (0.13 ± 0.02 vs. 0.08 ± 0.02 nmol/L, *p* = 0.037). 

### 3.6. Substrate Oxidation and Turnover

**Figure 4:**
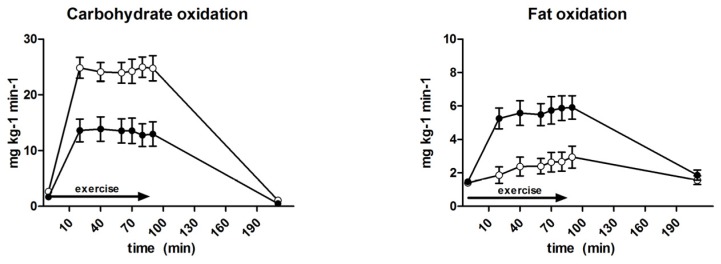
Carbohydrate (CHO) and fat oxidation during GLUFRU (black circle) and GLU (white circle). Results are expressed as mean ± SEM. Left to right: CHO oxidation, *p* < 0.001; fat oxidation, *p* < 0.001.

Substrate oxidation is outlined in Figure 4 and Table 2. Fat (1.5 ± 0.1 and 1.6 ± 0.2 mg·kg^−1^·min^−1^, *p* = 0.69) and CHO (1.7 ± 0.5 and 2.5 ± 0.3 mg·kg^−1^·min^−1^, *p* = 0.10) oxidation at baseline were comparable between GLUFRU and GLU. During the 90 min exercise period, fat oxidation was significantly higher and CHO oxidation lower in GLUFRU compared to GLU (5.2 ± 0.2 vs. 2.6 ± 1.2 mg·kg^−1^·min^−1^ and 18.1 ± 0.8 mg·kg^−1^·min^−1^ vs. 24.5 ± 0.8 mg·kg^−1^·min^−1^, *p* < 0.001 for both). Respiratory exchange ratio was lower during GLUFRU compared to GLU (0.86 ± 0.01 vs. 0.93 ± 0.00, *p* < 0.001). Fat oxidation contributed to 45.8% ± 1.8% of overall energy production in GLUFRU, and 25.5% ± 1.4% in GLU (*p* < 0.001). Energy yield contribution from CHO oxidation was 54.2% ± 1.8% in GLUFRU and 74.8% ± 1.4% in GLU (*p* = 0.02). Fat (1.9 ± 0.21 vs. 1.7 ± 0.2 mg·kg^−1^·min^−1^, *p* = 0.41) and CHO (0.5 ± 0.3 vs. 1.2 ± 0.4 mg·kg^−1^·min^−1^, *p* = 0.09) oxidation were comparable 120 min after exercise completion between GLUFRU and GLU.

There was no significant difference in glucose turnover between GLUFRU and GLU during the last 30 min of exercise (Table 2). R_a_ was 7.0 ± 0.4 vs. 7.3 ± 0.4 mg·kg^−1^·min^−1^ (*p* = 0.53) in GLUFRU and GLU, respectively. R_d_ and MCR were comparable between GLUFRU and GLU (7.8 ± 0.3 vs. 7.6 ± 0.5 mL·kg^−1^·min^−1^ and 6.0 ± 0.3 vs. 5.9 ± 0.4 mL·kg^−1^·min^−1^; *p* = 0.57 and *p* = 0.80, respectively). Estimated muscle glycogen oxidation was significantly lower in GLUFRU compared to GLU (10.2 ± 0.9 vs. 17.5 ± 1.0 mg·kg^−1^·min^−1^, *p* < 0.001).

Gluconeogenesis from fructose in GLUFRU was 0.9 ± 0.1 mg·kg^−1^·min^−1^, contributing to 12.1% ± 1.1% of total Ra. Plasma fructose concentration in the last 30 min of exercise in GLUFRU was 154.7 ± 8.4 μmol/L.

^13^C-isotopic enrichment in breath samples showed exogenous fructose and glucose oxidation rates of 1.5 ± 0.1 mg·kg^−1^·min^−1^ in GLUFRU and 3.0 ± 0.3 mg·kg^−1^·min^−1^ in GLU, in concordance with the 50% lower administered amount of ^13^C-labeled CHO in GLUFRU when compared to GLU (0.09 vs. 0.19 g). 

## 4. Discussion

The metabolic and hormonal effects of two different CHO supplementation approaches during exercise in patients with type 1 diabetes under identical insulin levels were compared in this study: glucose-fructose co-ingestion (GLUFRU) and glucose alone (GLU). Although the total amount of exogenous CHO needed to maintain stable glycaemia during the whole exercise period did not differ significantly, there was a tendency towards lower CHO requirements during GLUFRU (approximately 50% lower amounts compared to GLU) within the last 30 min of exercise (primary outcome). In daily clinical settings, this may have practical implications as the frequency of CHO supplementation could be reduced when using GLUFRU, thereby potentially providing convenience and safety to the exercising individual with type 1 diabetes.

GLUFRU resulted in significantly higher fat oxidation and lower CHO oxidation, and this was related to lower muscle glycogen oxidation in GLUFRU. Although identical exercise protocols were followed, GLUFRU increased lactate levels, suggesting that ingested fructose was partially converted into lactate. Thus, the benefits of GLUFRU supplementation in exercising individuals with type 1 diabetes may be its more sustainable glycaemic effect, in conjunction with increased fat utilization and sparing of muscle glycogen. 

To our knowledge, this is the first study in type 1 diabetes to investigate the metabolic effects of fructose when used in combination with glucose as a CHO supplementation to maintain stable glycaemia during exercise. The observed higher fat oxidation in GLUFRU compared to GLU is in line with a previous study in type 1 diabetes comparing a single oral load of 75 g glucose with an equivalent amount of isomaltulose (a disaccharide of glucose and fructose linked by an alpha-1,6-glycosidic bond), covered with identical insulin boluses 2 h pre-exercise. The authors observed lower CHO oxidation and greater lipid oxidation when 45 min of treadmill running was performed at 80% VO_2max_ [10]. Most studies assessing the effects of glucose-fructose ingestion compared to glucose alone, however, were performed in healthy non-diabetic individuals [15,29]. These studies adopted notably higher CHO feeding rates than those used in the present study (up to 2.4 g/min vs. 0.45 g/min), to maximize CHO absorption, which is the limiting factor for glucose-only supplementation regimes [30]. The authors were able to confirm their hypothesis that glucose-fructose co-ingestion increased total CHO absorption, and consequently metabolic substrate availability, which translated to greater exogenous CHO oxidation and improved endurance performance. Findings related to net substrate oxidation have been inconsistent, however, with some showing either similar [14] or higher [15,31] CHO oxidation with glucose-fructose co-ingestion, whereas others showed tendency to lower CHO oxidation [16]. Direct comparison between type 1 diabetes and healthy non-diabetic individuals is challenging, as CHO intake elicits endogenous insulin secretion in the latter, which is the main determinant in fuel selection [32]. Therefore, studies in individuals with type 1 diabetes, if performed under standardized conditions with identical insulinemia, may offer a unique opportunity to investigate isolated metabolic effects of fructose in an exercise context.

Our finding of increased lactate levels for GLUFRU is in line with other studies reporting partial conversion of ingested fructose into lactate following first pass hepatic metabolism [33,34,35,36]. It has been well reported that lactate is readily oxidized by the working muscle [37], therefore fructose-derived lactate may provide an efficient fuel during exercise. This statement is supported by a study comparing glucose-fructose co-ingestion with glucose alone in exercising healthy non-diabetic individuals, which showed that fructose-derived lactate oxidation and fructose-derived glucose oxidation each accounted for approximately 50% of net fructose oxidation [15]. Notably, the present study observed a net ^13^C-fructose oxidation of 1.5 mg·kg^−1^·min^−1^ in conjunction with a gluconeogenesis from fructose of 0.9 mg·kg^−1^·min^−1^, suggesting that the difference (approximately 0.6 mg·kg^−1^·min^−1^) may be accounted for by fructose-derived lactate oxidation.

The findings of higher fat oxidation and lower CHO oxidation under GLUFRU in the present study were unexpected, given the comparable levels of insulin, blood glucose, and gluco-regulatory hormones. The CHO concentration (20% vs. 10% solutions) and administration regime (tendency towards lower CHO supply in GLUFRU within last 30 min of exercise) were the only accountable differences between GLUFRU and GLU. The resulting differences in CHO intake-related glucose appearance is further compounded by the two-step metabolisation of fructose, which requires initial conversion into primary energy substrates such as glucose and lactate, for it to be used as fuel in the working muscle. 

Could one therefore hypothesise that the observed increased fat oxidation may be related to an increased utilization of fructose-derived lactate? Continuous low rate of fructose-derived lactate oxidation through lactate dehydrogenase reaction increases the NADH/NAD+ ratio, which then may lower pyruvate dehydrogenase (PDH) activity, thereby favouring acetyl-CoA production by β-oxidation [38]. At a certain level of β-oxidation, associated energy provision becomes self-sustaining as the generated acetyl-CoA and NADH further diminish PDH activity [39]. Testing this hypothesis, however, is beyond the scope of the present work and would need further investigation. Despite differences in fat oxidation, NEFA levels were comparable between GLUFRU and GLU. Such a constellation (higher fat oxidation despite comparable NEFA levels) has also been reported by others [10,40] and may suggest that differences in fat utilization may be attributable to intramyocellular lipid (IMCL), rather than peripheral adipose tissue lipolysis. This is further supported by the well-known fact that in comparison to peripheral lipolysis, intramyocellular hormone-sensitive lipase is not suppressed by the relatively high and constant exogenous insulin levels in our study population [41]. However, we acknowledge that the lack of IMCL assessment in the present study precludes statements regarding intramyocellular lipid utilization.

Interestingly, dopamine levels were significantly higher in GLUFRU compared to GLU in the present study. Due to the standardized setting dietary effects are unlikely to have contributed to this this finding. Although it is known that renal tubular cells can metabolize fructose [42], there is no data suggesting that this could alter the clearance of dopamine leading to higher circulating levels. We acknowledge that the observed difference in dopamine may entirely be due to chance. Although there is limited evidence suggesting dopamine to increase fat oxidation, further studies are needed to validate this hypothesis [43,44]. 

Post-exercise glycogen repletion has been shown to be related to a decline in blood glucose levels and, consequently, an increased risk for hypoglycaemia in exercising individuals with type 1 diabetes [45]. Therefore, the higher fat oxidation and glycogen-sparing effect observed in GLUFRU may be particularly beneficial for these individuals, pointing towards a potential role of fructose as a supplementary CHO for exercising individuals with type 1 diabetes. Of note, concerns have emerged related to adverse metabolic effects of fructose, albeit in sedentary individuals with relatively high intake of fructose-enriched diet [46,47,48]. These adverse metabolic effects have been shown to be potentially reversed by physical activity [49,50]. This is in line with the present findings that oxidation is the predominant metabolic fate of ingested fructose which thereby serves as an efficient fuel under exercising conditions.

The strengths of the present study are its standardized design, which comprehensively combines independent techniques to investigate exercise-related fuel metabolism in a commonly encountered clinical situation (e.g., performing exercise without adjustment of insulin doses). The adoption of the study intervention in daily practice is feasible, and potentially provides metabolic benefits to exercising individuals with type 1 diabetes. 

Due to the complexity of the chosen technical approach, this study has several limitations, and therefore our results will need to be interpreted with caution. The non-significant difference of the primary endpoint may have been related to the relatively small sample size, and the variability in carbohydrate absorption. In addition, the study had a non-randomised design, and the exclusive recruitment of well-controlled male participants may have limited the generalizability of our findings. The latter was to mitigate against metabolic differences related to sex hormones [51,52]. Larger randomised studies will be needed to validate findings from the present study. The tracer calculations used were based on a one compartment model [53], which may not be fully appropriate for non-steady state conditions. Muscle glycogen oxidation may have been underestimated if less than 100% of glucose R_d_ was oxidised. However, the probability that a major amount of glucose may have been oxidised in non-muscle tissue under exercise conditions is low. Of note, the metabolic fate of co-ingested glucose could not be determined during GLUFRU, as only fructose was labelled. Additionally, for logistical reasons the amount of fructose disposed though systemic lactate was not measured by our tracer methods, as this would have required duplication of tests for each participant to separately monitor glucose and lactate kinetics.

## 5. Conclusions

In conclusion, the present study shows that GLUFRU is equally effective as GLU in stabilizing glycaemia in type 1 diabetes during exercise, and induces a shift towards higher fat oxidation with concomitant glycogen-sparing effect in the working muscle. These findings corroborate the flexibility of exercise-related fuel metabolism in type 1 diabetes, indicating that glucose-fructose co-ingestion may be a promising strategy to optimise fuel metabolism during exercise in type 1 diabetes. Further studies are needed to explore the related mechanisms in more detail, such as evaluating different fructose intake doses and schedules under reduced insulin doses, as per clinical guidelines [4].

## Figures and Tables

**Table 1:** Baseline characteristics. Data presented as mean (SD).

	*n* = 15
Age (years)	26.1 ± 4.8
Weight (kg)	80.4 ± 10.7
Height (m)	1.81 ± 0.08
BMI (kg/m^2^)	24.5 ± 3.2
Fat-free mass (%)	78.8 ± 7.1
BMR (MJ/day)	8.3 ± 0.9
VO_2max_ (mL·(kg·body·weight)^−1^·min^−1^)	47 ± 9
Diabetes duration (years)	13.3 ± 6.7
Haemoglobin A1c (%)	7.0 ± 0.6
Haemoglobin A1c (mmol/mol)	53 ± 7
Total average daily insulin (U·kg^−1^·day^−1^)	0.7 ± 0.1

BMI = body mass index. BMR = basal metabolic rate. VO_2max_ = maximal oxygen uptake.

**Table 2:** Metabolic measures during last 30 min of exercise in GLUFRU and GLU. Values are mean (SEM). GLUFRU = glucose-fructose co-ingestion, GLU = glucose alone ingestion.

	GLUFRU (*n* = 15)	GLU (*n* = 15)	*p* Value
Carbohydrate requirements (g)	34.0 ± 2.9	37.8 ± 5.3	0.46
Carbohydrate oxidation (mg·kg^−1^·min^−1^)	18.1 ± 0.8	24.5 ± 0.8	<0.001
Fat oxidation (mg·kg^−1^·min^−1^)	5.2 ± 0.2	2.6 ± 1.2	<0.001
Glucose appearance, R_a_ (mg·kg^−1^·min^−1^)	7.0 ± 0.4	7.3 ± 0.4	0.53
Glucose disappearance, R_d_ (mg·kg^−1^·min^−1^)	7.8 ± 0.3	7.6 ± 0.5	0.57
Metabolic clearance rate, MCR (mg·kg^−1^·min^−1^)	6.0 ± 0.3	5.9 ± 0.4	0.80
